# Chinese massage (Tuina) combline with paraffin therapy versus tuina or paraffin therapy alone for the treatment of congenital muscular torticollis: A protocol for systematic review and Bayesian network meta-analysis

**DOI:** 10.1097/MD.0000000000027648

**Published:** 2021-11-05

**Authors:** Haikuo Song, Xuan Zhang, Xiangfa Zou

**Affiliations:** a Department of Orthopedics and Traumatology of Integrated Traditional Chinese and Western Medicine, Tianjin Hospital, Tianjin, China; b College of Acupuncture, Tianjin University of Traditional Chinese Medicine, Tianjin, China; c Department of Integrated Traditional Chinese and Western Medicine, the Second Hospital of Dalian Medical University, Liaoning, China.

**Keywords:** congenital muscular torticollis, meta, paraffin therapy, protocol, review, tuina

## Abstract

**Background:**

Current studies in patients with congenital muscular torticollis (CMT) have predominantly focused on the role of tuina or paraffin therapy alone. This systematic review with Bayesian network meta-analysis will be performed to sum up the existing evidence on the effects and safety of tuina plus paraffin therapy for CMT in infants and children.

**Methods:**

The Preferred Reporting Items for Systematic Reviews and Meta-Analyzes reporting guidelines will be followed to conduct this study. The electronic databases of PubMed, Cochrane Library, PsycINFO, EMBASE, the Chinese Scientific Journal Database, China National Knowledge Infrastructure, WanFang Data, Taiwan Electronic Periodical Services, and Web of Science will be searched from the inception to November 2021 using the following key terms: “Tuina,” “traditional Chinese medicine massage,” “paraffin,” and “congenital muscular torticollis,” for all relevant studies. We impose no language restrictions. We include reports on randomized controlled trials (RCTs) and quasi-RCTs of Tuina combline with paraffin therapy for the treatment of CMT in children and adolescents. We include studies that assessed effective rate, symmetry, improvements of range of motion, muscle length, and sternocleidomastoid tumor thickness, quality of life, and adverse events. The Cochrane Bias Risk Tool, which considers sequence generation, allocation concealment, and blinding and other aspects of bias, will be used to assess the risk of bias in studies.

**Results:**

A Bayesian network meta-analysis is an appropriate statistical method to compare all treatment options by statistically simulating the estimated results of a comprehensive trial, and to compare treatments by common and associated comparators. In addition, Bayesian network meta-analysis can produce ranking probabilities of treatments, which may contribute to clinicians’ clinical decision-making.

**Registration number:**

10.17605/OSF.IO/K5EGN.

## 1 Introduction

Congenital muscular torticollis (CMT) is characterized by a cervical deformity caused by excessive contraction or shortening of the sternocleidomastoid muscle, with or without a palpable mass, and its pathogenesis is thought to be related to muscular trauma at birth or chronic repetitive microtrauma, such as chronic intrauterine poor posture.^[1]^ During the diagnosis of CMT in infants, physical therapy examinations are performed to assess passive and active neck rotation and lateral flexion, craniofacial asymmetry, and head/skull shape. The goal of the treatment is to provide unlimited range of neck motion, balanced neck muscle function, and symmetrical midline head position to prevent plagiocephaly and facial asymmetry.^[2]^

Massage is usually thought of as a form of complementary and alternative therapy in which the practitioner manipulates the surface of the recipient's body. In general, massage therapy has 3 beneficial effects: emotional effects, which are related to emotions and feelings; physiological effects, relating to the basic processes of the organism; and behavioral effects, which are associated with people's reactions to circumstances.^[3]^ Traditional Chinese medicine massage, also known as tuina, is based on the meridian theory of Chinese medicine and aims to stimulate specific acupoints or meridians on the surface of the body to achieve therapeutic effects.^[4]^ Pediatric tuina stimulates the meridians and acupoints to regulate yin and yang, order descent and ascent, tonify the deficient functions, purge the excess functions to restore health, and generate warming and clearing effects.^[5]^ A recent review of randomized controlled trials (RCTs) suggests that massage therapy may have beneficial effects on treating CMT in infants and children.^[6]^

Paraffin therapy is a form of heat therapy that has become widely used in hospitals as a physical treatment for patients with musculoskeletal problems. Paraffin therapy provides surface heat to muscle and tendon tissue and relaxes the smooth muscle fibers in the arterioles, improving local circulation.^[7]^ Previous studies have found that paraffin therapy can reduce pain and improve range of motion in people with arthritis of the hand.^[8,9]^ However, to our knowledge, few studies have examined the effects of paraffin therapy on CMT in children.

Current studies in patients with CMT have predominantly focused on the role of tuina or paraffin therapy alone.^[6,10]^ The safety of tuina combined with paraffin therapy is largely unknown and must be determined by a systematic review. We aim to fill this research gap by providing the latest evidence for researchers, doctors, and patients. This systematic review with Bayesian network meta-analysis will be performed to sum up the existing evidence on the effects and safety of tuina plus paraffin therapy for CMT in infants and children.

## 2 Materials and methods

### 2.1 Search methods for the identification of studies

The systematic review protocol has been registered on Open Science Framework registries (10.17605/OSF.IO/K5EGN). The Preferred Reporting Items for Systematic Reviews and Meta-Analyzes reporting guidelines will be followed to conduct this study. The electronic databases of PubMed, Cochrane Library, PsycINFO, EMBASE, the Chinese Scientific Journal Database, China National Knowledge Infrastructure, WanFang Data, Taiwan Electronic Periodical Services, and Web of Science will be searched from the inception to November 2021 using the following key terms: “Tuina,” “traditional Chinese medicine massage,” “paraffin,” and “congenital muscular torticollis,” for all relevant studies. For Chinese databases, equivalent Chinese group terms will be searched. We impose no language restrictions. Furthermore, the reference lists from published original articles and relevant reviews will be assessed to identify more relevant studies. Ethical approval is not necessary because the present meta-analysis will be performed on the basis of previous published studies.

### 2.2 Eligibility criteria

#### 2.2.1 Types of studies

We include reports on RCTs and quasi-RCTs of tuina combline with paraffin therapy for the treatment of CMT in children and adolescents.

#### 2.2.2 Types of subjects

We include studies that targeted children and adolescents aged 18 years or younger who have been diagnosed with CMT. We will not exclude the studies if the participants have other comorbidities.

#### 2.2.3 Types of intervention

Patients receiving with tuina plus paraffin therapy will be regarded as intervention group.

#### 2.2.4 Types of comparison

Patients treated with tuina alone or paraffin therapy alone will be considered as the control group

#### 2.2.5 Types of outcome measures

We include studies that assessed effective rate, symmetry, improvements of range of motion, muscle length, and sternocleidomastoid tumor thickness, quality of life, and adverse events.

Retrospective studies, biomechanical studies, in vitro studies, review articles, techniques, case reports, letters to the editor, and editorials will be excluded.

### 2.3 Data collection and risk of bias assessment

Qualified RCTs identified from our search efforts will be screened by 2 investigators and validated by a third investigator on the team to confirm that each study meets our eligibility criteria. Using predesigned forms, which will initially be piloted in a small number of inclusion studies, the same reviewers will also be responsible for extracting and validating data on general features of inclusion studies, feature-related interventions, outcomes and study design. If the results are not reported at a predefined time point, we will extract data as close to that time point as possible.

The Cochrane Bias Risk Tool, which considers sequence generation, allocation concealment, and blinding and other aspects of bias, will be used to assess the risk of bias in studies. The overall risk of bias rating for each study will be the lowest rating for any criteria (e.g., if any domain is scored high risk of bias, the study will be considered high risk of bias). Any disagreement between reviewers on any of the above steps will be discussed by the reviewers until consensus is reached. Authors of major publications, collaborators, and/or sponsors of clinical trials will also be contacted for missing outcome data or unclear information.

### 2.4 Statistical analysis

We use WinBUGS 1.4.3 software (MRC Biostatistics Unit, Cambridge, UK) and NetMetaXL (Canadian Agency for Drugs and Technologies in Health, Ottawa, Canada) to conduct a Bayesian network meta-analysis. Network meta-analysis combines data from several different randomized comparisons of different treatments to provide an internally consistent set of estimates, while respecting randomization in each trial. The network meta-analysis will be performed within a generalized linear model framework with link functions that specify the relationship between the results and the model coefficients to be estimated. When the outcome is continuous, the likelihood will be modeled as normal. When the outcome is the event rate, the likelihood will be modeled as Poisson. The random effects model will be used for this analysis. Estimation will be performed in a Bayesian context using the noninformation prior distribution of the parameters. The model will be evaluated using the Deviation Information Criterion, a measure that combines model fit and complexity. The analysis will be estimated using a Bayesian Markov Chain Monte Carlo model.

## 3 Discussion

Current studies in patients with CMT have predominantly focused on the role of tuina or paraffin therapy alone. The safety of tuina combined with paraffin therapy is largely unknown and must be determined by a systematic review. We aim to fill this research gap by providing the latest evidence for researchers, doctors, and patients. This systematic review with Bayesian network meta-analysis will be performed to sum up the existing evidence on the effects and safety of tuina plus paraffin therapy for CMT in infants and children. A Bayesian network meta-analysis is an appropriate statistical method to compare all treatment options by statistically simulating the estimated results of a comprehensive trial, and to compare treatments by common and associated comparators. In addition, Bayesian network meta-analysis can produce ranking probabilities of treatments, which may contribute to clinicians’ clinical decision-making.

## Author contributions

**Conceptualization:** Xuan Zhang.

**Data curation:** Haikuo Song, Xuan Zhang.

**Formal analysis:** Haikuo Song, Xuan Zhang.

**Funding acquisition:** Xiangfa Zou.

**Investigation:** Haikuo Song, Xuan Zhang.

**Methodology:** Xuan Zhang.

**Project administration:** Xiangfa Zou.

**Resources:** Xiangfa Zou.

**Software:** Haikuo Song.

**Supervision:** Xiangfa Zou.

**Writing – original draft:** Haikuo Song.

**Writing – review & editing:** Xiangfa Zou.
